# Adsorption in a Fixed-Bed Column and Stability of the Antibiotic Oxytetracycline Supported on Zn(II)-[2-Methylimidazolate] Frameworks in Aqueous Media

**DOI:** 10.1371/journal.pone.0128436

**Published:** 2015-06-09

**Authors:** Janine dos Santos Ferreira da Silva, Daniel López Malo, Giovana Anceski Bataglion, Marcos Nogueira Eberlin, Célia Machado Ronconi, Severino Alves Júnior, Gilberto Fernandes de Sá

**Affiliations:** 1 Departamento de Química Fundamental, Universidade Federal de Pernambuco, Recife, Pernambuco, Brasil; 2 Instituto de Química, Universidade Estadual de Campinas, Campinas, São Paulo, Brasil; 3 Instituto de Química, Universidade Federal Fluminense, Nitéroi, Rio de Janeiro, Brasil; NERC Centre for Ecology & Hydrology, UNITED KINGDOM

## Abstract

A metal-organic framework, Zn-[2-methylimidazolate] frameworks (ZIF-8), was used as adsorbent material to remove different concentrations of oxytetracycline (OTC) antibiotic in a fixed-bed column. The OTC was studied at concentrations of 10, 25 and 40 mg L^-1^. At 40 mg L^-1^, the breakthrough point was reached after approximately 10 minutes, while at 10 and 25 mg L^-1^ this point was reached in about 30 minutes. The highest removal rate of 60% for the 10 mg L^-1^ concentration was reached after 200 minutes. The highest adsorption capacity (28.3 mg g^-1^) was attained for 25 mg L^-1^ of OTC. After the adsorption process, a band shift was observed in the UV-Vis spectrum of the eluate. Additional studies were carried out to determine the cause of this band shift, involving a mass spectrometry (MS) analysis of the supernatant liquid during the process. This investigation revealed that the main route of adsorption consisted of the coordination of OTC with the metallic zinc centers of ZIF-8. The materials were characterized by thermal analysis (TA), scanning electron microscopy (SEM), powder X-ray diffraction (XRD), and infrared spectroscopy (IR) before and after adsorption, confirming the presence of OTC in the ZIF-8 and the latter’s structural stability after the adsorption process.

## Introduction

Studies of environmental impacts caused by chemical pollution usually focus on conventional pollutants such as dyes, heavy metals and carcinogenic substances [1–4]. However, several tons of pharmaceutical drugs are synthesized every year for human and veterinary use, and are considered contaminants of emerging concern [5–7]. Although the toxic effects of drugs have not yet been fully investigated, various studies have shown that the accumulation of these substances may interfere with the metabolism of aquatic organisms, resulting in an increase in bacterial resistance and its potential risk to health [8–11].

Antibiotics deserve special attention because of their intensive use in human and veterinary medicine and in agriculture [12–14]. The antibiotic oxytetracycline (OTC) belongs to the class of tetracyclines and its mechanism of action is the inhibition of bacterial protein synthesis [15]. OTC is being prescribed for diseases such as sinusitis, bronchitis, cholera, acne, urinary tract infections, leptospirosis, relapsing fever, and others.

The human body absorbs from 60 to 80% of oxytetracycline [16]. This drug is considered the most important Antibiotic Growth Promoter, used in animal breeding programs and is often found at fish farms and swine feeding facilities [17–20]. One concern is that a considerable fraction of 20 to 90% is not absorbed by the animal and is excreted unaltered [17].

Antibiotics enter aquatic environments through industrial wastewater that passes unaltered through treatment facilities and sewage treatment plants, since conventional techniques such as chemical coagulation, sedimentation, digesters, and other disinfection techniques have limited ability to remove variety drugs [21, 22].

A strategy to remove antibiotics from effluents at sewage treatment plants, after a traditional treatment is to include a solid phase adsorption step. Recently, the use of bamboo charcoal to adsorb tetracycline and chloramphenicol in aqueous solutions has been reported [23].

A new route using inorganic porous materials which is under development involves the so-called “three dimensional coordination networks,” also known as “Metal Organic Frameworks” (MOFs), which are polymeric networks of metallic centers with organic ligands [24–26]. Their stability in water, large surface area and accessible pore structure are properties that indicate the suitability of MOFs as good adsorbent materials in aqueous media [27, 28].

The use of some Metal Organic Frameworks has already proved effective in removing pollutants from wastewaters. MOF-235, iron terephthalate, has been used in the adsorptive removal of methyl orange and methylene blue from aqueous solutions [29]. The MOF MIL-53(Al) is considered an efficient adsorbent for the removal of nitrobenzene from aqueous solutions [30]. The MOF MIL-101(Cr) was applied successfully in the adsorption of xylenol orange [31]. And the MOFs MIL-101(Fe), MIL-100 (Fe), HKUST-1([Cu_3_(TMA)_2_(H_2_O)_3_]*n*) and MIL-100(Cr) have been studied for the adsorption of drugs in wastewater [32,33].

**Fig 1:**
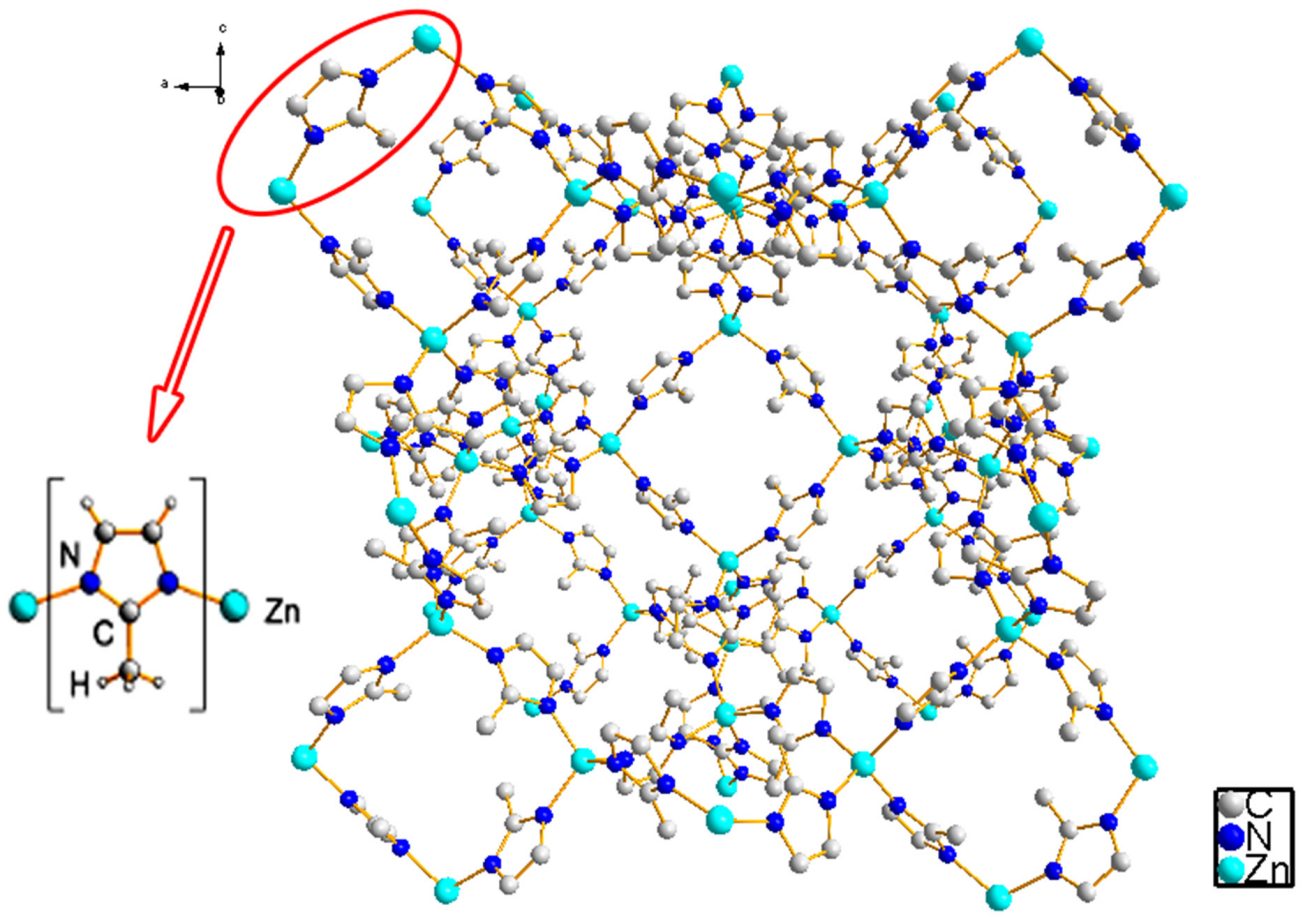
ZIF-8 structure.

The MOF used in this work belongs to the family of Zeolitic Imidazolate Frameworks (ZIF) [34, 35]. Specifically, this group possesses tetrahedral metal centers, commonly Zn (II) or Co (II), which are coordinated by nitrogen atoms in positions 1 and 3 of the ligand imidazolate, C_3_N_2_H_3_^-^. ZIF materials combine the thermal stability of zeolites with the broad structural diversity, harmony, and pore size congruence of Metal Organic Frameworks [36, 37]. The ZIF-8 [34] used in this study consisted of Zn (II) metal centers coordinated by the ligand 2-methylimidazole (Fig 1).

Conspicuously, MOFs coordinated by the ligand imidazolate exhibit network topologies corresponding to the sodalite type zeolites that are widely employed as successful adsorbent materials [38, 39]. Zeolitic Imidazolate Frameworks number 8 offers high porosity, vast surface area and good stability in water [33, 35, 36, 37, 40]. All these properties make ZIF-8 an effective aqueous adsorbent material. Furthermore, this material has recently shown good interaction with tetracycline antibiotics in chromatographic analysis as a solid phase adsorbent material [41].

The present work involved a study of the fixed-bed column adsorption process of the antibiotic oxytetracycline in an aqueous medium, using the coordination network ZIF-8 as adsorbent material. The material was characterized after the adsorption process in order to identify the adsorption mechanism, and the possible degradation of both OTC and ZIF-8 was analyzed by mass spectrometry.

## Materials and Methods

### Reagents and experimental apparatus

All the reagents used here were analytically pure unless stated otherwise. Solutions were prepared in water purified by reverse osmosis and then deionized (18MΩcm) with a Milli-Q gradient Millipak 40.

ZIF-8 acquired from BASF (Aldrich) under the commercial name Basolite Z1200 was used with no previous treatment. Its BET surface is 1300–1800 m^2^g^-1^, particle size 4.9 μm, pore size 0.6 nm, physical density without guest molecules is 0.95 g cm^-1^, porosity 58.8% and the pore volume is 0.663 cm^3^ g^-1^. [36, 40].

The oxytetracycline hydrochloride C_22_H_24_N_2_O_9_.HCl, (95% purity), HPLC grade was supplied by Aldrich.

Other chemicals employed were HCl (Vetec), NaOH (Dinâmica) to adjust the pH, methanol (Dinâmica), acetonitrile (Cromoline), MgSO_4_ (Vetec), and Al(NO_3_)_3_ (Vetec) for the desorption process.

The pH of the solutions was measured with the aid of a Quimis Q400MT pH meter.

**Fig 2:**
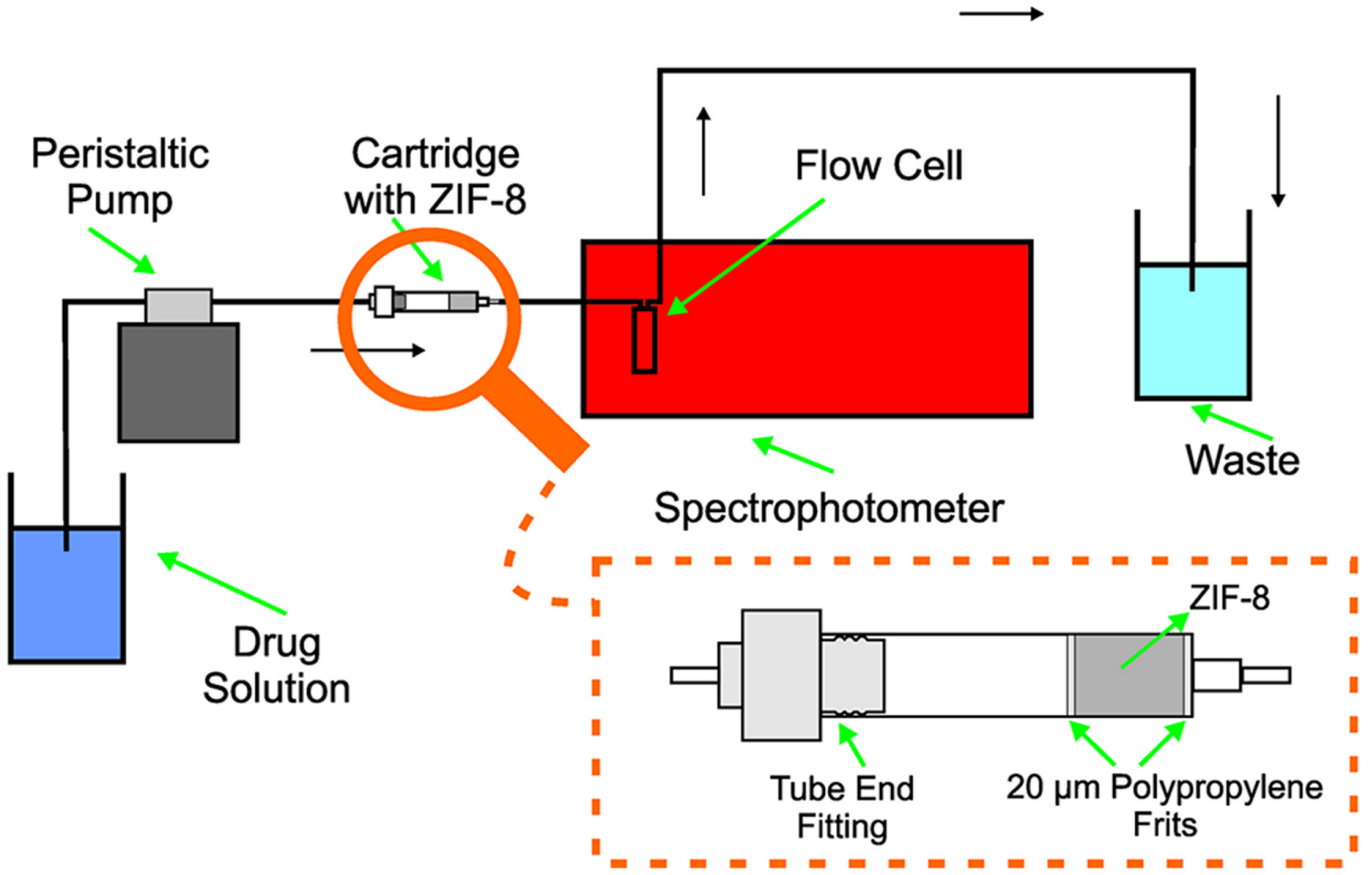
Manifold employed for online fixed-bed adsorption.

The adsorption studies were performed in columns using the flow manifold depicted in Fig 2. The apparatus consisted of a Gilson MINIPULS 3 peristaltic pump with Tygon pump tubing (Worthington, OH, USA), which delivered the drug solution to the adsorbent material placed in an empty 1 mL Agilent Bond Elut solid phase extraction cartridge attached to a low-pressure tube end fitting. Finally, the apparatus was connected to an Ionlab Q-76 quartz flow cell and placed in a Perkin Elmer Lambda 650 spectrophotometer. All the connections were made with 0.8 mm i.d. PTFE coil.

## Procedures

### Preparation of standards

Standard solutions of oxytetracycline were prepared by weighing the exact amount of antibiotic for 10, 25 and 40 mg L^-1^ solutions, which was dissolved in deionized water with the aid of an ultrasonic bath. The solutions had a pH of 4.3.

During the adsorption process, the oxytetracycline concentration was determined from the absorbance at 353.35 nm with the aid of an external calibration curve with standard solutions (0.1–40 mg L^-1^).

### Fixed-bed column adsorption process

Fifty mg of ZIF-8 were placed in the solid phase extraction cartridge for all the adsorption assays. The internal diameter of the cartridge was 5.75 mm and after being filled with the adsorbent material had a height of 11 mm.

**Fig 3:**
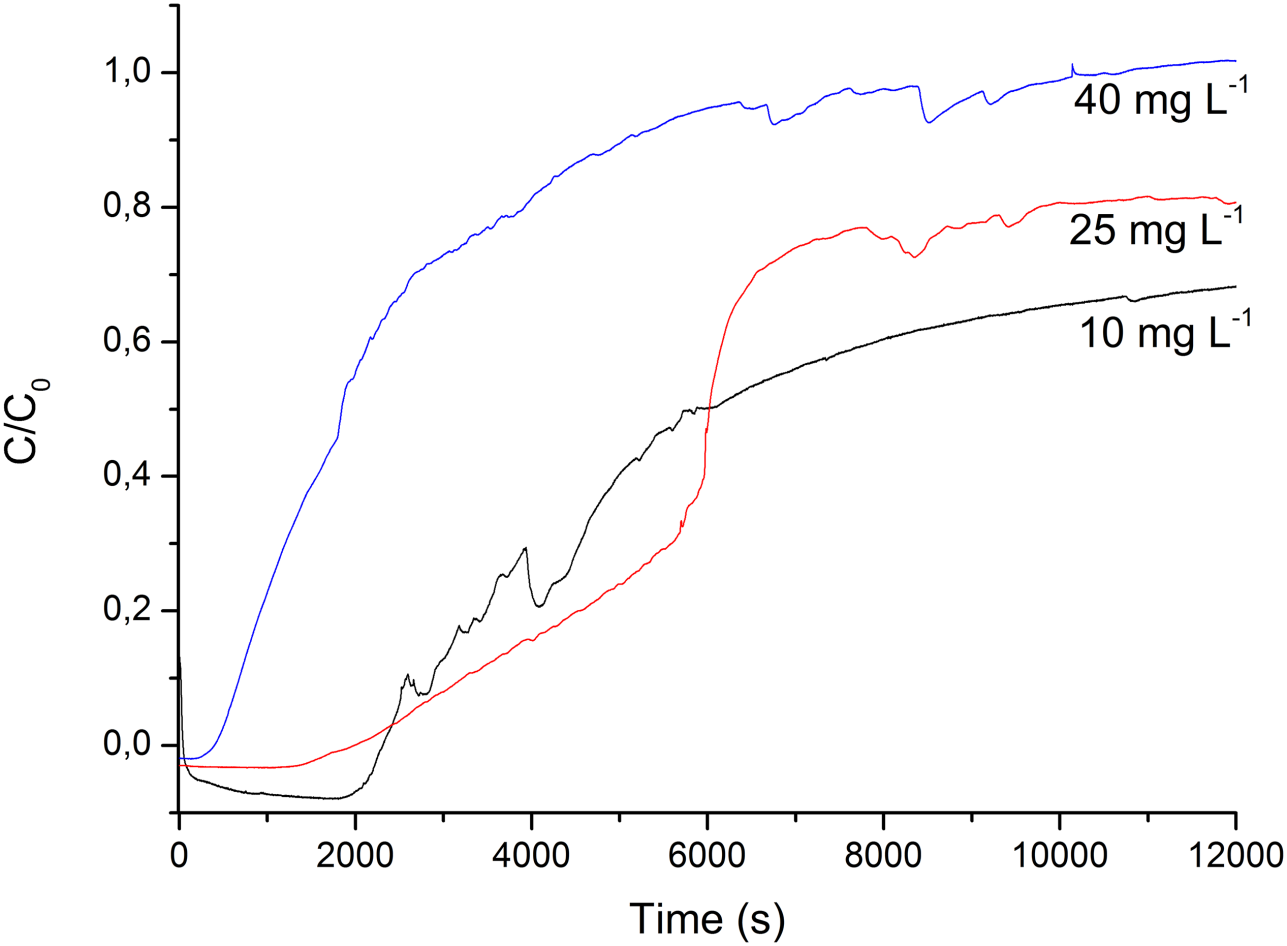
Breakthrough curves of the continuous flow adsorption processes at the three tested OTC concentrations: 10, 25 and 40 mg L^-1^.

The oxytetracycline solution was passed through the manifold (Fig 3) for 200 minutes at a flow rate of 0.5 mL min^-1^. The assays were performed at room temperature (~25°C).

To evaluate the adsorption of the fixed-bed column, breakthrough curves were studied by plotting the relative amount of antibiotic eluted (C/C_0_) as a function of time [42].

The total amount of antibiotic (q_total_) removed during the continuous flow was calculated from the integrated area under the breakthrough curve described by the oxytetracycline concentration removed (C_R_) as a function of time, as described in Eq 1 [43], where C_R_ is the concentration of removed antibiotic (mg L^-1^) calculated from the difference between the initial concentration of oxytetracycline in the effluent and the concentration of oxytetracycline in the equilibrium after a time t and Q is the flow rate (mL min^-1^).

$$q_{total}=\;\frac{Q}{1000}\overset{t_{total}}{\underset{0}{\int }}C_{R}dt$$ (1)

The total amount of adsorbate (m_total_), in mg, that passed through the column is given by Eq 2,

$$m_{total}=\;\frac{C_{i}.Q.t_{total}}{1000}$$ (2)

where C_i_ is the initial concentration of the antibiotic solution and t_total_ is the analysis time.

The percentage of removed antibiotic (%R) was calculated from the ratio of q_total_ to m_total_ given by Eq 3,

$$\%R=\;\frac{q_{total}}{m_{total}}\;x\;100.$$ (3)

The amount of adsorbed material in the equilibrium state or adsorbent adsorption capacity q_e_ (mg of adsorbed antibiotic/g of adsorbent), and the equilibrium concentration of adsorbate, C_e_ (mg L^-1^), were calculated using Eqs 4 and 5, respectively,

$$q_{e}=\frac{q_{total}}{m}$$ (4)

where m is the mass of adsorbent expressed in g,

$$C_{e}=\frac{m_{total}-q_{total}}{V_{efluent}}\;x\;1000$$ (5)

### Characterization of ZIF-8 before and after the adsorption process

Initially, both oxytetracycline and ZIF-8 were characterized prior to the adsorption process. After 24 hours of contact time by batch adsorption, consisting on adsorbent material in direct contact with a given volume of contaminant solution under agitation, until equilibrium was reached, with the purpose of a more homogeneous contact with the adsorbent. The resulting material was filtered, dried, and characterized by the techniques described below in order to study alterations in the structure of the adsorbing material and the oxytetracycline.

The process was performed at three oxytetracycline concentrations: 10, 25 and 40 mg L^-1^. A UV-Vis analysis indicated the absence of the drug in the supernatant solution.

Another batch adsorption assay was then performed using 20 mg of ZIF-8 and 50 ml of 25 mg L^-1^ OTC solution for the mass spectrometry study. The OTC adsorption in MOF was monitored using a first generation Synapt HDMS (High Definition Mass Spectrometry; Waters Corp., Manchester, UK). This mass spectrometer is equipped with an ESI source and has a hybrid quadrupole/ion mobility/orthogonal acceleration time-of-flight geometry (oa-TOF).

Aliquots of the reaction mixture were collected after each given time, centrifuged, and the supernatant solution subjected to ESI (+). The conditions of this source were as follows: capillary voltage 3.0 kV, sample cone 30 V, extraction cone 3 V, source temperature 100°C, desolvation temperature 100°C, and desolvation flow rate 300 mL min^-1^ of N_2_. For the MS experiments, trap and transfer cells were operated at 6 and 4 V, respectively. For the MS/MS experiments, the ions of interest were selected in the quadrupole analyzer, fragmented in the transfer cell using argon as collision gas (the transfer energy was optimized for each ion of interest), and the product ions analyzed by TOF.

### Physical Measurements

The samples were prepared on carbon tape on an aluminum support and coated with a 10–20 nm gold film, using a Bal-Tec SCD 050 sputter coater. Images were recorded by a scanning electron microscope (Jeol JSM-5900 SEM) operating at a voltage of 15 KV, a 4.0 probe and a working distance of 18 to 24 nm. Diffractograms were recorded at room temperature in the range of 5° to 50° in a Rigaku 2400 DMAX X-ray diffractometer (XRD) with Cu Kα (0.15 nm). The thermogravimetric analyses were performed in a Shimadzu DTG-60H analyzer in a N_2_ atmosphere from room temperature to 800°C (10°C min^-1^). Vibrational spectra were obtained a Bruker IFS 66v/S FTIR spectrometer in the range of 4000–400 cm^-1^.

Pore volume and surface area measurements were performed in a Micromeritics ASAP 2010 apparatus. About 100 mg of each sample was dried in an oven at 100°C for 12 h, before they were introduced in a quartz cell and attached to the physisorption apparatus. The sample was dried under vacuum at 100°C for 5 h. The BET surface area and pore volume were obtained by N_2_ physisorption at 77.4 K.

## Results and Discussion

### Fixed-bed column adsorption process

A breakthrough curve was obtained for each tested oxytetracycline concentration, and the absorbance was measured at one-second intervals for a total of 200 minutes after the fixed bed became saturated (Fig 3).

The antibiotic removal rate was about 100% efficient for a period of 30 minutes in the assays using 10 and 25 mg L^-1^ OTC solutions. After that period, an amount of more than 5%, the breaking point, was detected by UV-VIS in the eluate. The best performance was achieved in these assays.

It was found that more than 5% of the initial concentration of oxytetracycline was adsorbed at the beginning of the adsorption process of the 40 mg L^-1^ concentration, indicating that the breaking point was reached very quickly.

The total amount of OTC removed (q_total_) in 200 minutes in the different assays was calculated using the integration of the area under the curve described by the oxytetracycline concentration removed (C_R_) as a function of time, shown in the Table 1.

**Table 1:** Breakthrough curve fixed-bed column adsorption parameters of the antibiotic OTC on ZIF-8.

Assay	Ci(mg L^-1^)	q_total_(mg)	m_total_(mg)	R(%)	q_e_(mg g^-1^)	C_e_(mg L^-1^)
1	10	0.60	1.0	60.0	12	4.0
2	25	1.42	2.5	56.6	28.3	10.8
3	40	0.83	4.0	20.7	16.6	31.7

The breakthrough curve parameters were obtained using Eqs 1 to 5 given in the experimental section.

The removal of oxytetracycline using ZIF-8 varied from 20.7 and 60% for a period of 200 minutes of treatment depending on the initial OTC concentration. The lower OTC concentrations showed a better adsorption yield, using the same amount of adsorbent material and the same flow rate in all the assays.

Assays 1 and 2 resulted in a significant OTC removal rate in relation to the total mass of OTC that passed through the column (m_t_). Assay 3 showed a lower OTC removal rate, i.e., R(%) = 20.7, which can be explained by the high concentration of antibiotic used in this assay. However, OTC concentrations found in the environment and described in the literature [44, 45] are at μg L^-1^ levels. Our purpose in using higher OTC concentrations was to achieve a broader level of adsorbed antibiotic and to ensure proper adsorption at lower concentrations.

After contact with ZIF-8, the pH of the eluate increased from 4.0 to 9.0. This increase may be attributed to an ion exchange with the medium. Oxytetracycline exists mainly as a zwitterion positively charged in the tertiary amine and negatively charge in the deprotonated hydroxyl group, which derives from intramolecular proton transfer [46] at pH levels of 4 to 8. This may be a determining factor in defining intermolecular and intramolecular interactions due to the influence of both pH and ionic strength.

### Study of the possible degradation of oxytetracycline after contact with ZIF-8

**Fig 4:**
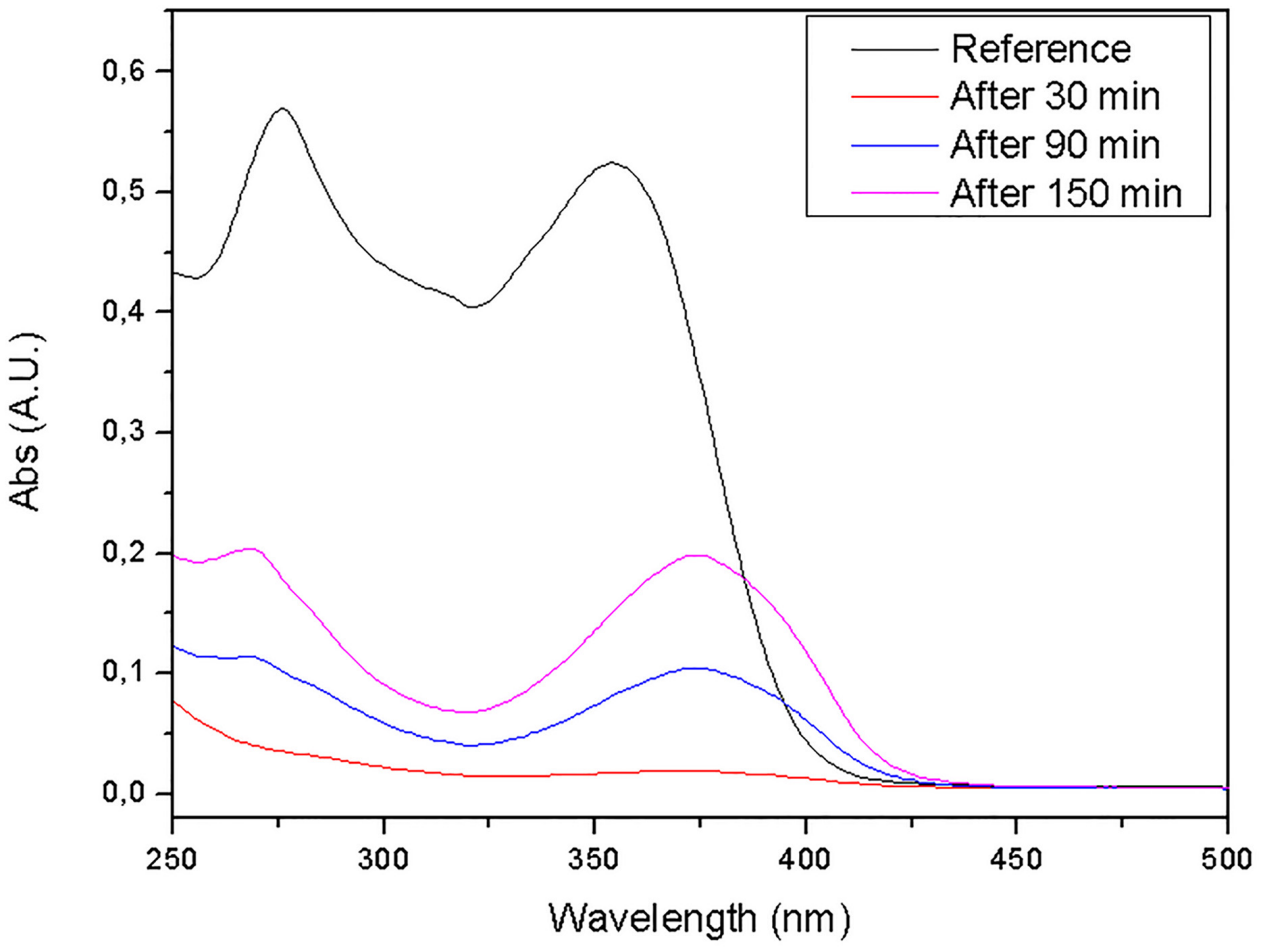
UV-VIS absorption spectra of OTC reference solution (10 mg L^-1^) in deionized water and eluates collected at different contact times with ZIF-8 to study possible antibiotic degradation.

After the continuous flow adsorption process was completed, the UV-Vis absorption band of the eluate showed a wavelength shift of approximately 20 nm, with the wavelength of maximum absorbance shifting from 353.35 to 374 nm, as shown in Fig 4. Despite the band shift observed on the oxytetracycline absorption spectrum, the absorbance readings at fixed wavelength were interpolated using the external calibration curve. Moreover, when the adsorbent material is saturated, the stock solution concentration is found on the readings of the eluate, making sense operate this way.

**Fig 5:**
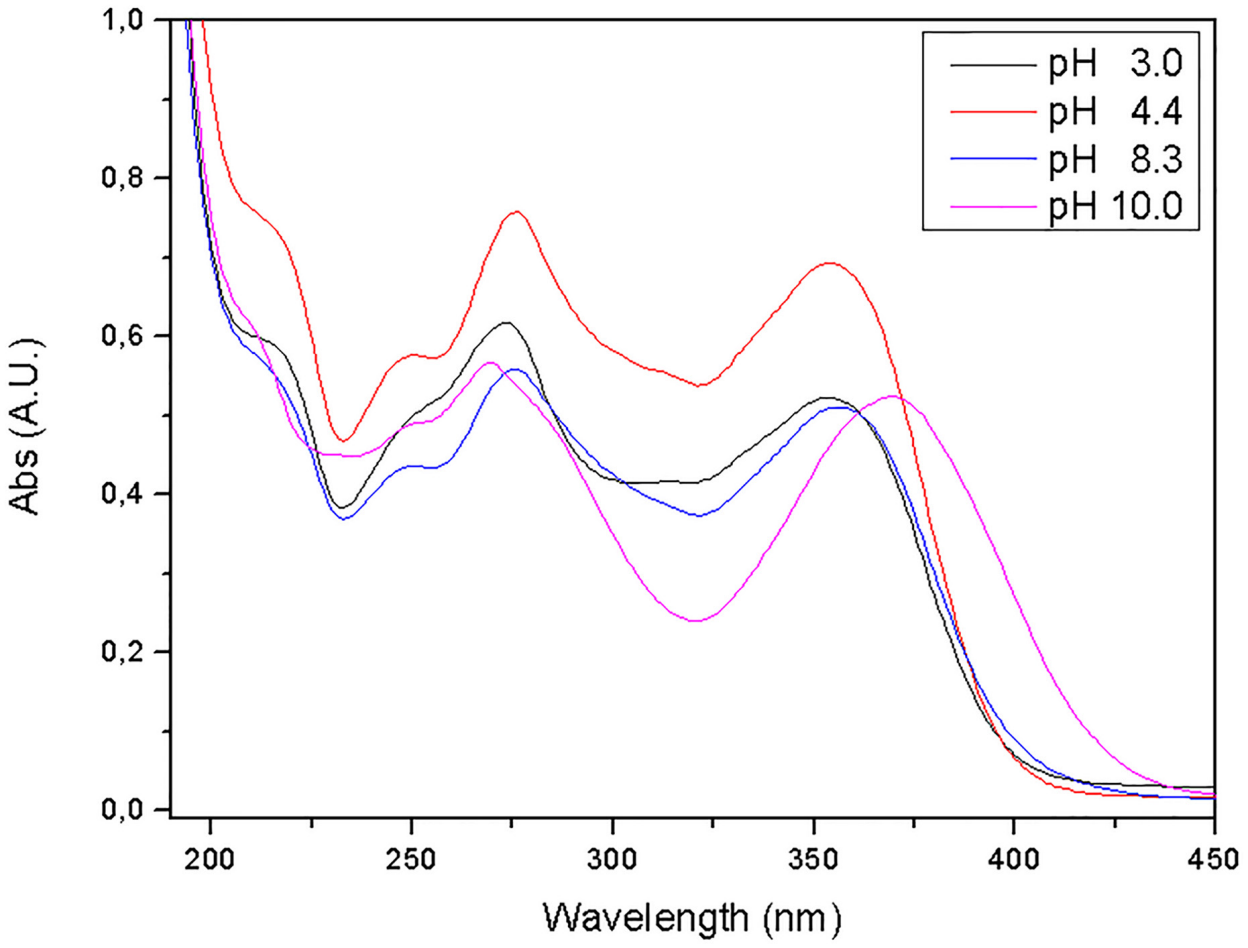
UV-Vis absorption spectra of OTC (25 mg L^-1^) in deionized water at different pH values.

The Bathochromic band shift was studied by changing the pH of the oxytetracycline solution. UV-VIS absorbance spectra were then recorded to establish a correlation between the increase in pH and the band shift of the antibiotic **(** Fig 5).

A direct correlation can be observed between the increase in the pH of the oxytetracycline solution and the band shift [47, 48]. However, the magnitude of this shift was not the same as the one observed using ZIF-8.

**Fig 6:**
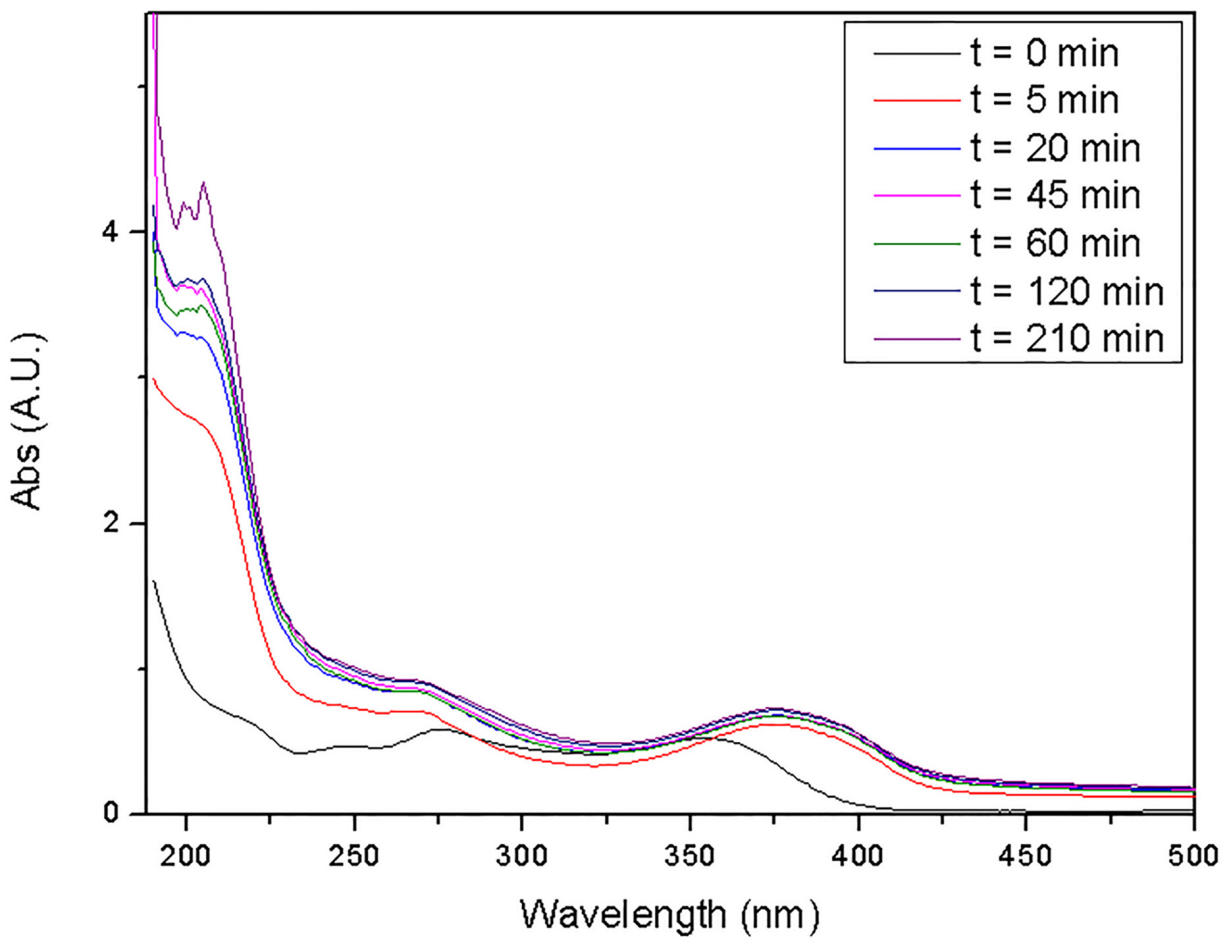
UV-Vis absorption spectra of OTC aliquots obtained in deionized water at different contact times with ZIF-8.

Another batch adsorption process was performed to determine if the shift appears gradually. Aliquots of the supernatant solution were collected and analyzed by UV-Vis at different times, i.e., 5, 20, 45, 60, 120 and 210 minutes. As can be seen in Fig 6, the shift had already occurred at the 5 minute measurement, but no further shift was recorded after longer periods of contact with ZIF-8.

**Fig 7:**
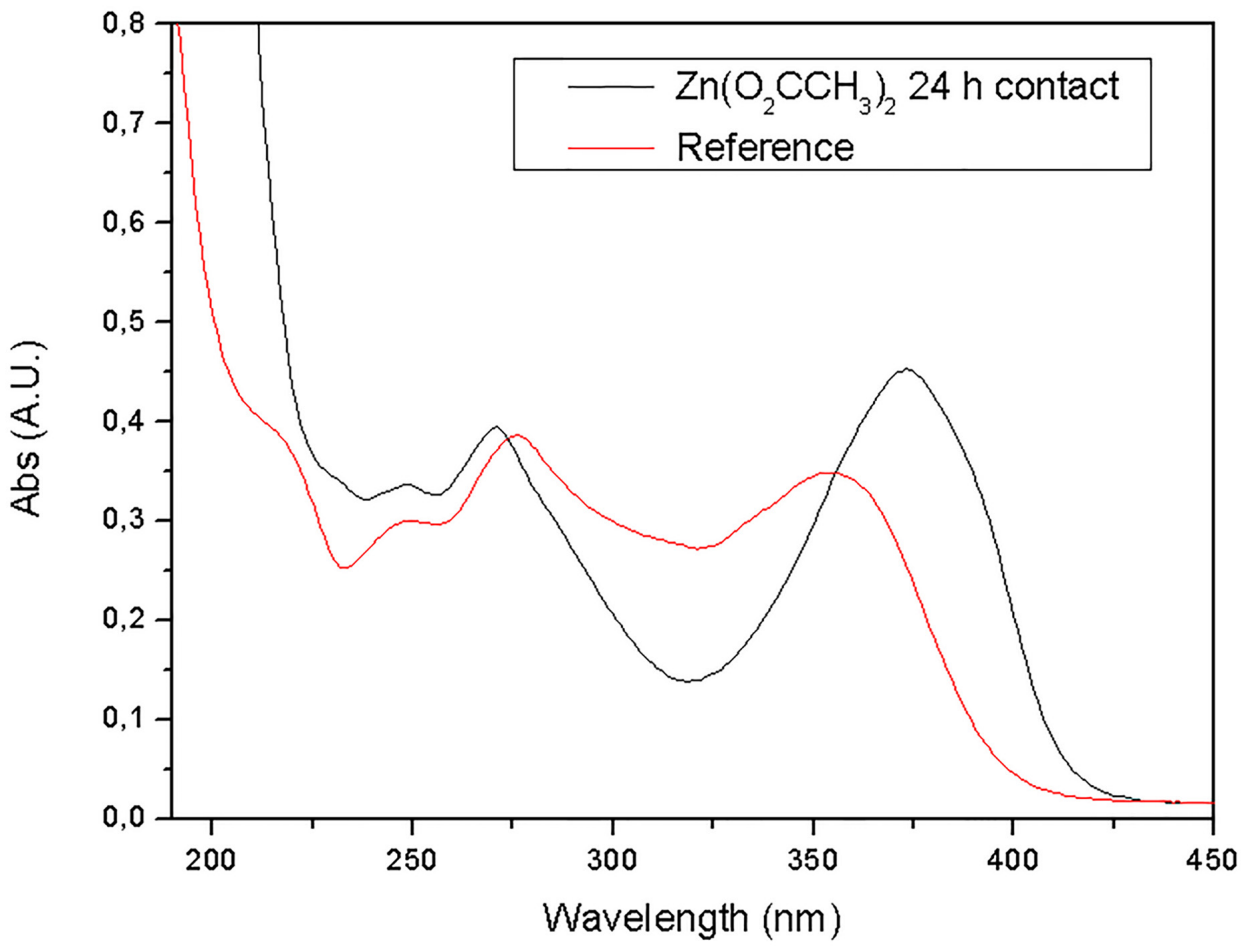
UV-Vis absorption spectra of OTC aliquots of reference and after contact with Zn(CH_3_COO)_2_ for 24h.

Another batch assay was performed to examine the interaction between zinc and the antibiotic OTC. To this end, a solution of zinc acetate was mixed with 50 mL of a 25 mg L^-1^ OTC solution. The UV-Vis spectrum was then recorded and compared with the reference OTC solution. As can be seen in Fig 7, a bathochromic band shift occurred from 353.35 to 374 nm, suggesting that the oxytetracycline molecules on coordination with the d orbitals of the Zn^2+^, form the ligand-to-metal charge-transfer (LMCT) coordination compound, during the adsorption process [49].

**Fig 8:**
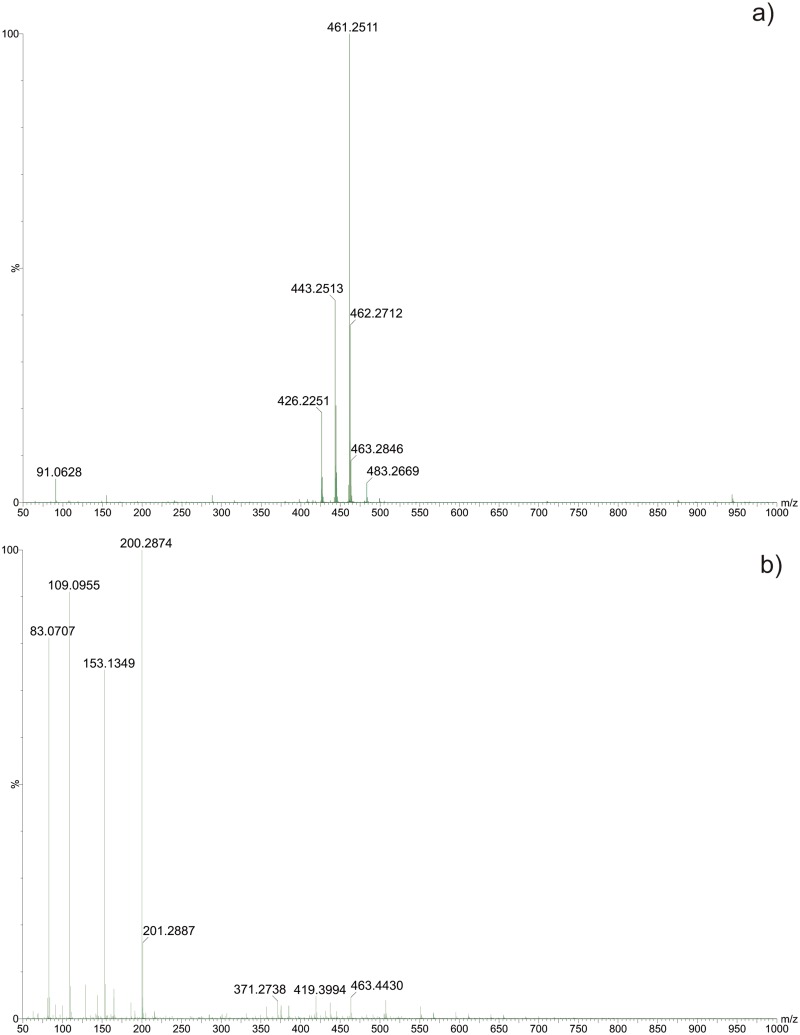
ESI(+)-MS of (A) Oxytetracycline, and (B) suspension of ZIF-8 supernatant.

In view of the results of the above described experiments, an MS analysis was performed to determine whether ZIF-8 was coordinating or degrading the antibiotic. Firstly, mass spectra were recorded of both ZIF-8 and oxytetracycline, whose characteristic ions are shown in Fig 8.

The oxytetracycline mass spectra show ions corresponding to protonated molecules, sodium adducts and ions characteristic of NH_3_ and H_2_O loss. Mass spectra of the ZIF-8 supernatant suspension reveal the ion at *m/z* 83, which is related to 2-methylimidazole (ligand of ZIF-8), and other unidentified ions.

After characterizing the OTC and ZIF-8, the adsorption process continued, with aliquots removed at 3, 5, 15, 30, 60, 90 and 120 minutes for ESI-MS analysis.

**Fig 9:**
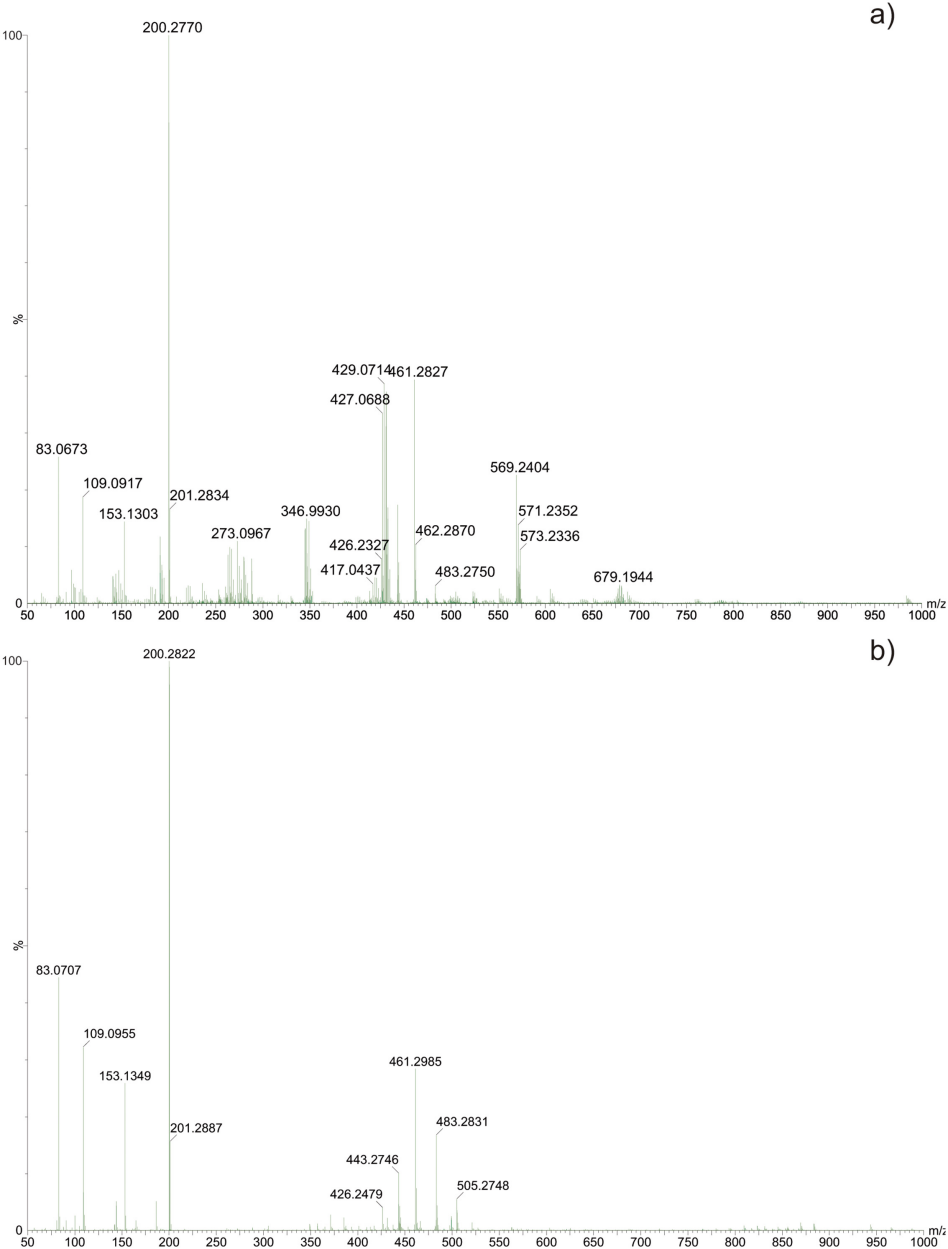
ESI(+)-MS of supernatant liquid aliquot: (A) after 3 minutes, and (B) after 120 minutes of contact between ZIF-8 and OTC.

Fig 9 shows representative mass spectra of two analyzed aliquots.

The mass spectra recorded during the adsorption process show the same ions as those observed in the oxytetracycline and ZIF-8, but no ions related to OTC degradation products were detected. Two new ions were observed at the onset of adsorption, the first one at *m/z* 505 and the second at *m/z* 569. The appearance of the ion at *m/z* 505 is explained by the association of the deprotonated OTC [M–H] with sodium ions, [M–H + Na_2_]^+^. Eventually, OTC was also coordinated by free zinc from ZIF-8, [M–H + Na_2_ + Zn]^+^, generating the corresponding ion at *m/z* 569, where the presence of zinc was confirmed by the isotopic pattern. The analysis of the next aliquots revealed a decrease in the intensity of the ions corresponding to the OTC, indicating the occurrence of the adsorption process on ZIF-8. In addition, the ion at *m/z* 569 disappeared, probably due to the lower amount of OTC available for coordination in the solution.

The MS analysis revealed the absence of degradation processes, suggesting that adsorption was the only phenomenon responsible for the decrease of oxytetracycline in solution. These associations between OTC and Zn present in the coordination network are responsible for the band shift observed in the UV-Vis absorption spectra of the OTC eluates.

Antibiotics are electron rich compounds, while metallic ions are electron deficient. As for their chemical structure, the tetracycline group is composed of molecules with donor groups (N, O), which are good complexing agents [50]. The attraction between these species with opposing signals makes them prone to interact, and the coordination compounds resulting from these interactions are usually quite stable [51–53]. The literature contains several reports about the coordination of antibiotics with a metal to overcome bacterial resistance; however, the use of coordination chemistry for the adsorption of pollutants in wastewater is an innovative strategy.

The UV-Vis spectrum of the oxytetracycline solution reveals four absorption bands centered at 360, 314, 265 and 218 nm. According to McCormick et al. [52], the π-π* transition of the first ring of the tricarbonyl group containing the amine group contributes only to the 260 nm band. However, the π-π* transition of the chromophore group that is present in the remaining rings contributes to all the bands, particularly to the band centered at around 360 nm. These transitions are sensitive to deprotonation and complexation, i.e., the addition of a coordinating metal noticeably changes the absorption spectrum.

In the work of McCormick et al. [54], the major modification involved in the absorption corresponded to the chromophore formed by benzene ring conjugated carbonyl and to enol present in oxytetracycline, which displayed a band shift from 354 to 374 nm. This suggests, therefore, the involvement of this chromophore in the OTC coordination with the Zn present in the ZIF-8 structure.

### Characterization before and after the adsorption process

#### Infrared spectroscopy (IR)

The intense stretching band in oxytetracycline is centered between 3400 and 3300 cm^-1^ due to the O–H from alcohol and phenolic groups. The C = O band partially overlaps the N–H band, which appears between 1640 cm^-1^ and 1620 cm^-1^, appearing as a small doublet. The characteristic aromatic ring vibrations appear at around 1450 cm^-1^, which is consistent with reports in the literature [55, 56].

In the IR spectrum of ZIF-8 containing the 2–methyl–imidazole ligand, the N–H stretching band is visible at 3135 cm^-1^ and the C–H stretching bands of the methyl groups at 2929 (imidazole aliphatic C–H) and 1307 cm^-1^. The band corresponding to C–N and C = N is located at 1457, 1423 and 1145 cm^-1^, and is displaced due to coordination with the metallic center. An axial deformation is visible in the C = N band at 1576 cm^-1^. The absorption band at 421 cm^-1^ is related to Zn–N stretching. The arrangement of the bands is in agreement with that expected for the organic ligand, and similar results are reported in the literature [57].

**Fig 10:**
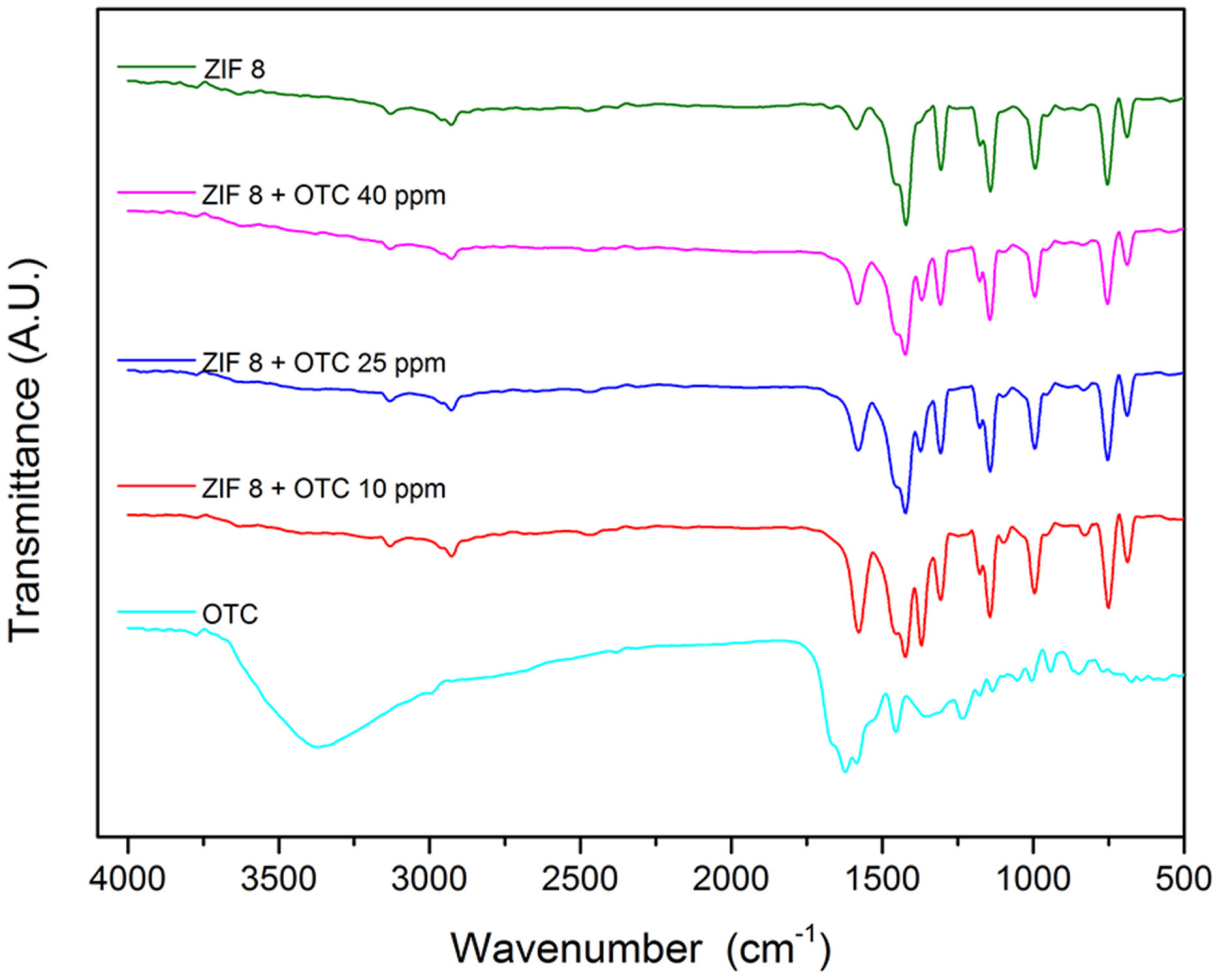
Overlapped IR spectra of ZIF-8, OTC, and the product of adsorption at different tested concentrations.

After the adsorption process of oxytetracycline in ZIF-8, the resulting solids were analyzed by IR spectroscopy. Fig 10 compares the above described starting materials, and the products obtained in the different adsorption processes. According to the IR spectra, the chemical bonds show no visible changes. A slight increase can be noted in the axial deformation if the C = N band at 1576 cm^-1^. With regard to the OTC, the OH band from 3100 cm^-1^ to 3600 cm^-1^ disappeared, indicating deprotonation of the OH groups. The appearance of a new band at around 1300 cm^-1^ may be due to the C–O^-^ stretching band from OTC deprotonated oxygen atoms.

The IR spectra also showed no OTC carbonyl groups, and the amide band that appeared previously at 1630 cm^-1^ (ν_C = O_) was also missing in the product of the adsorption process, indicating coordination with metallic ions [58].

#### Scanning Electron Microscopy (SEM)

See S1 File.

#### Powder X-Ray Diffraction (XRD)

Powder X-ray diffraction was performed to examine the sample’s crystallographic phases qualitatively, as well as to evaluate possible alterations in the ZIF-8 structure after its contact with oxytetracycline.

**Fig 11:**
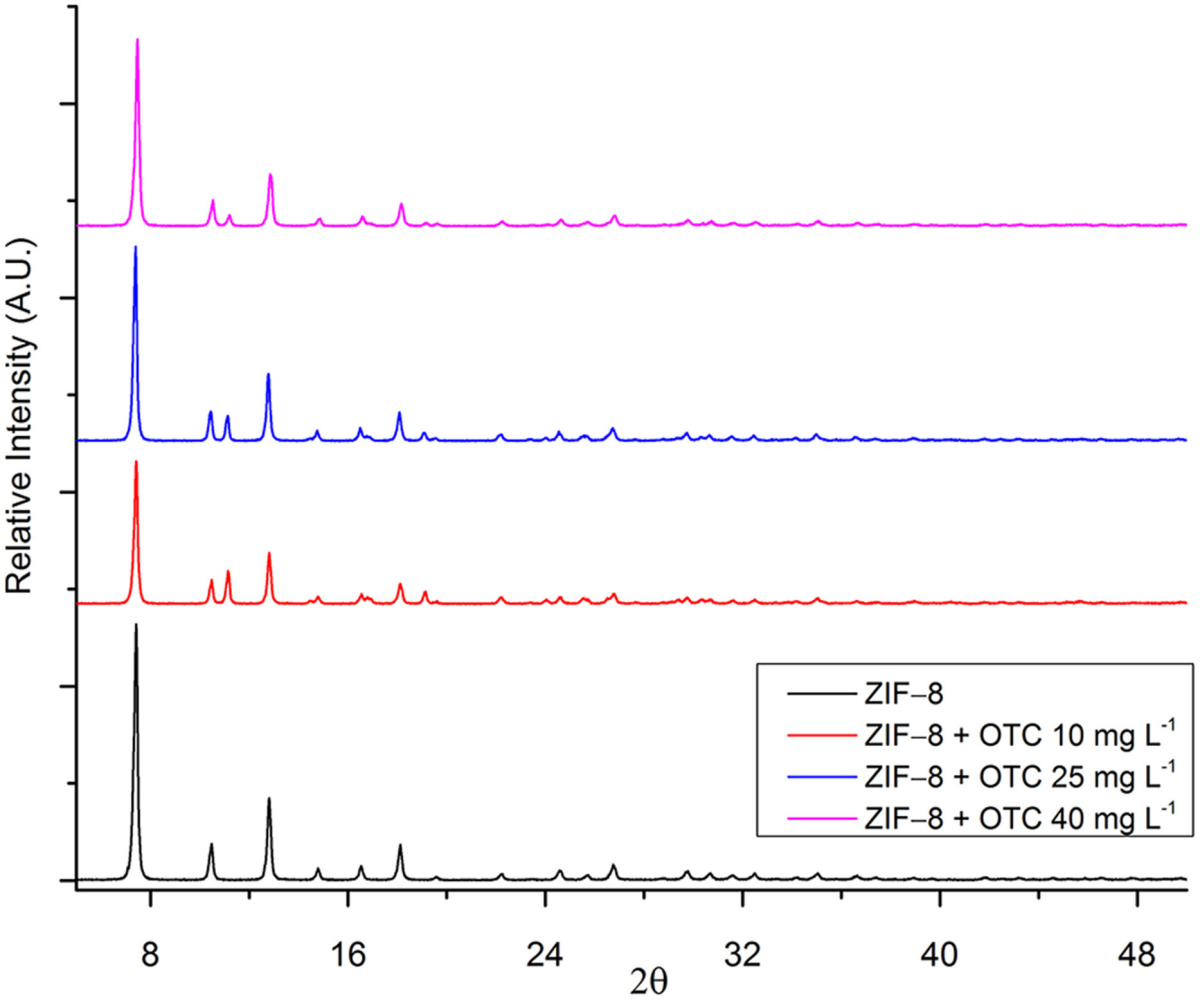
Overlapping diffractograms of ZIF-8 and the different adsorption products after contact with OTC.

Fig 11 shows the diffractograms of the ZIF-8 and the material resulting from the mixture of ZIF-8 and OTC at the concentrations of 10, 25 and 40 mg L^-1^ compared with the diffractogram of ZIF-8. The XRD pattern of the ZIF-8, in which the presence of thin peaks indicates high crystallinity, is in good agreement with other studies reported in the literature [36, 57, 59]. All the diffractograms show the characteristic peaks of ZIF-8, although indications of the OTC structure at 2θ = 11.6° and 18.8° are visible.

#### Thermogravimetric analysis (TGA)

The stability and thermal decomposition of the samples were evaluated by TGA before and after the adsorption process.

The thermoanalytical profile of ZIF-8 displays a single step, which can be interpreted as the loss of the organic ligand, imidazolate, in a temperature range of 350 to 650°C. The ligand mass (minus a C atom) is 152 g mol^-1^, representing 66.4% of the total mass. The theoretical value is in good agreement with the experimental data. The missing C is combined with zinc to form ZnC, generating a residual mass, after loss of the ligand, which corresponds to 33.6% of the molecular mass of ZIF-8.

The thermoanalytical profile of oxytetracycline hydrochloride shows a loss of HCl at about 100°C, which corresponds to 7.4% of the total mass and is in good agreement with the experimental loss of 7.424%. The remaining mass corresponds to the skeleton of OTC, 460 g mol^-1^, 92.6% of the total mass. Decomposition was found to occur in two steps. This data is also consistent with the experimental result of 93.2%.

**Fig 12:**
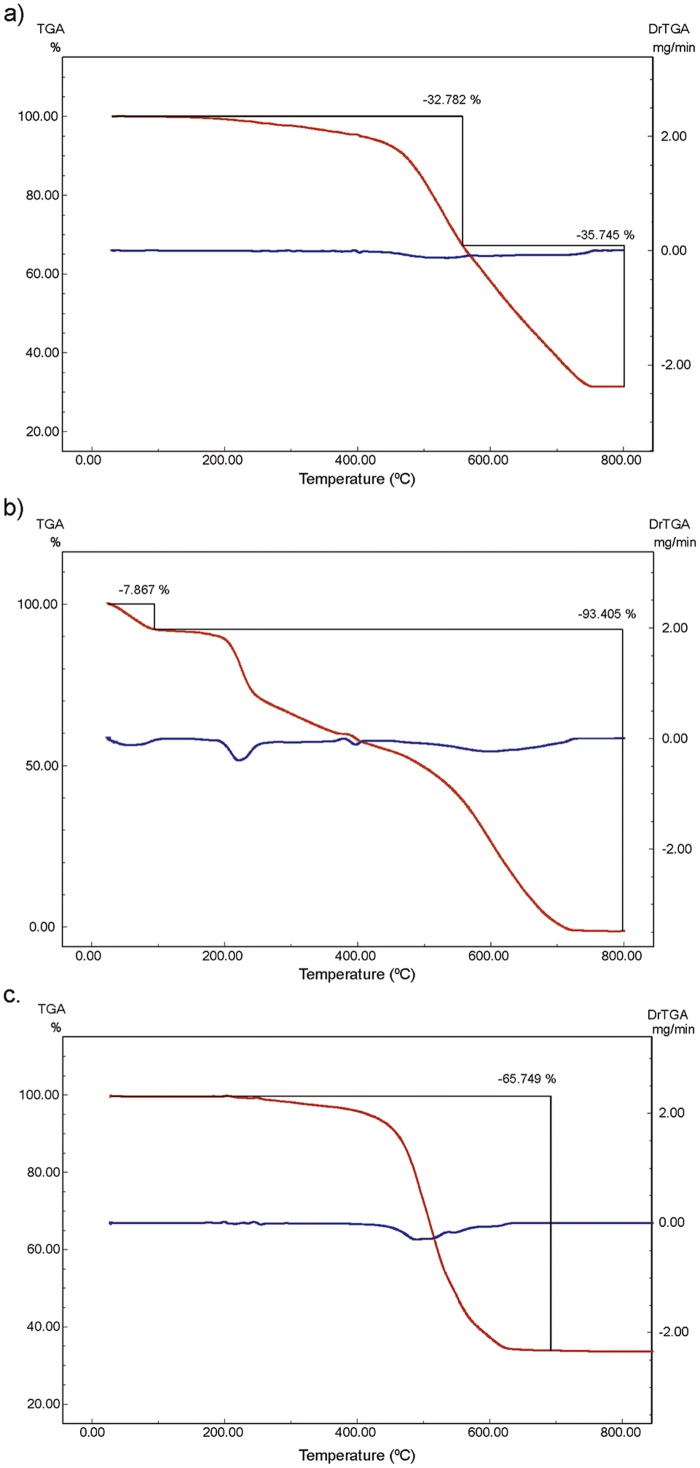
TGA of (a) adsorption product after contact with 25 mg L-1 OTC, (b) OTC and (c) ZIF-8.

Fig 12 shows the overlapping thermoanalytical profiles of OTC, ZIF-8 and the products of the adsorption process at 25 mg L^-1^ concentration of OTC. The other thermoanalytical profiles are depicted in Figure D in S1 File. Note that the compound resulting from the adsorption process showed higher thermal stability that the starting materials. The thermal decomposition started at about 450°C. The acquired stability of these compounds is described in the literature on bioinorganic chemistry [51, 52].

#### BET surface area analysis

**Fig 13:**
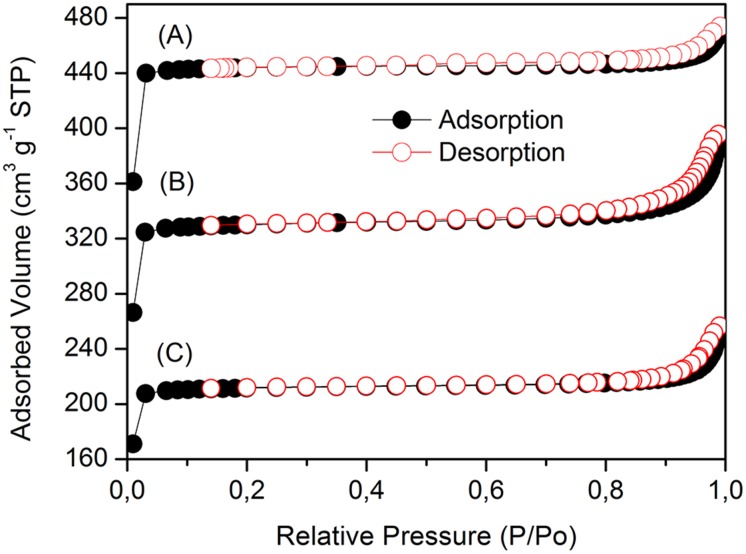
Nitrogen adsorption/desorption isotherms of (A) ZIF-8, (B) ZIF-8 + 10 mg L^-1^ of OTC and (C) ZIF-8 + 25 mg L^-1^ of OTC.

Fig 13 shows the N_2_ sorption isotherms of ZIF-8, ZIF-8 + 10 mg L^-1^ of oxytetracycline solution and ZIF-8 + 25 mg L^-1^ of OTC solution performed at 77.4 K. Typical Type I isotherms were obtained for all samples, which reveal their microporous nature [60]. However, the behavior of the isotherms for values of P/P_0_ > 0.95 changes to Type IV, which suggests the existence of large pores due to intra agregate voids.

The surface area and pore volume from the N_2_ adsorption isotherm of ZIF-8 are shown in Table 2. The BET and Langmuir surface area were 1745.4 and 1964.4 m^2^ g^-1^, respectively, and single point total pore volume = 0.720 cm^3^ g^-1^. These results are in good agreement with the literature [36].

**Table 2:** Surface area and pore volume for ZIF-8 and ZIF-8 loaded with different amounts of OTC.

Reference	BET surface area S_BET_/m^2^ g^-1^	Langmuir surface area SLang/m^2^ g^-1^	Single-point total pore volume Vp/cm^3^ g^-1^
**ZIF-8**	**1745.4**	**1964.4**	**0.720**
**ZIF-8 + 10 mg L** ^**-1**^ **OTC**	**1294.7**	**1458.2**	**0.590**
**ZIF-8 + 25 mg L** ^**-1**^ **OTC**	**831.8**	**935.0**	**0.377**

ZIF-8 loaded with 10 and 25 mg L^-1^ solutions of oxytetracycline showed a significant reduction of the surface area and pore volume (Fig 13 and Table 2, indicating an interaction between ZIF-8 and OTC molecules. The interesting structural features of ZIF-8, i.e. high surface area, large spherical cavities (11.8 Å), small apertures (3.4 Å) and structural flexibility make this material attractive for adsorption studies [36]. It has been revealed that ZIF-8 acts as a molecular sieve for xylene isomers [61]. Pirngruber *et al*. [61] showed that *para*-xylene, which the kinetic diameter (0.67 nm) is larger than the pore aperture (0.34 nm) of ZIF-8 can adsorb into it. Apparently, the pores open as a gate to allow the diffusion of the molecules into the cage of ZIF-8. Conversely, the adsorption of *ortho*-xylene (kinetic diameter = 0.74 nm) into the pores of ZIF-8 was insignificant and mainly occurred onto its external surface. Because OTC molecule is much larger than *ortho*-xylene, most likely OTC molecules are adsorbed onto the external surface of ZIF-8 instead of being inside of the pores.

## Conclusions

In this study, a methodology was developed to minimize the amount of oxytetracycline in aquatic media using ZIF-8 as adsorbent material, and its performance was considered satisfactory.

The adsorption process occurred by covalent bonding between the Zn metal present in the ZIF-8 and OTC, which resulted in a more stable compound than the raw materials. These findings were confirmed by the results of mass spectrometry and thermal analysis.

The removal of oxytetracycline was approximately 100% efficient for a period of 30 minutes, using antibiotic concentrations of 10 and 25 mg L^-1^. The results achieved in these conditions were better than those obtained with the OTC concentration of 40 mg L^-1^.

In the conditions used in this work, 50 mg of adsorbent material and a flow rate of 0.5 mL min^-1^, the percentage removal of oxytetracycline after 200 minutes varied from 20.7 to 60%, depending on the OTC initial concentration.

In view of the excellent adsorption behavior exhibited by the ZIF-8, the continuous system can be used as an integrated process for the treatment of wastewater contaminated with oxytetracycline.

## Supporting Information

S1 File Supporting information file which contains: SEM micrograph of commercial ZIF-8 (Figure A); SEM micrograph of OTC (Figure B), SEM micrograph of OTC 40 mg L^-1^ adsorbed on ZIF-8 (Figure C) and TGA of (a) adsorption product after contact with: 10 mg L^-1^ OTC and (b) 40 mg L^-1^ OTC (Figure D). (DOCX)
