# Supplementary material for: Adsorption in a Fixed-Bed Column and Stability of the Antibiotic Oxytetracycline Supported on Zn(II)-[2-Methylimidazolate] Frameworks in Aqueous Media
Source: PLoS One. 2015 Jun 9;10(6):e0128436. doi: 10.1371/journal.pone.0128436 (PMC4461271; doi:10.1371/journal.pone.0128436)
Supplement: S1 File — (DOCX) [file pone.0128436.s001.docx]

**Supporting Information**

**Scanning Electron Microscopy**

The surfaces and the composition of the samples before and after the adsorption process were evaluated qualitatively by SEM. The micrograph of commercial ZIF-8 shows small particles (see **Figure A**), possibly due to fast nucleation [57] during synthesis.

**
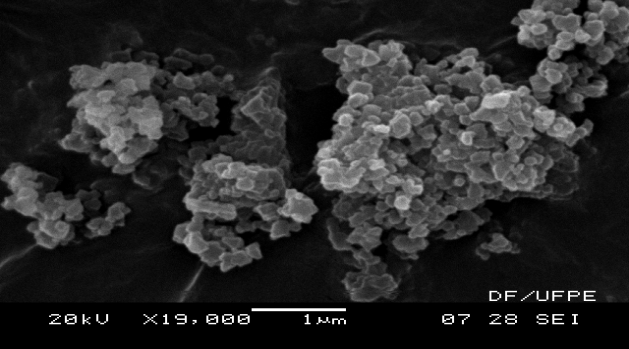
**

***Figure A.*** *SEM micrograph of commercial ZIF-****8.***

The micrograph of the OTC in **Figure B** reveals thin elongated thread-like particles of irregular lengths.

**
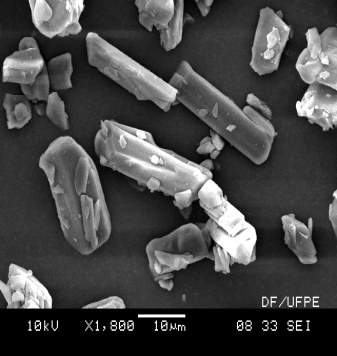
**

***Figure B.*** *SEM micrograph of OTC.*

The micrograph in **Figure C** shows the material obtained after the adsorption process using the 40 mg L^-1^ OTC solution, with the visible presence of the new coordination complex originated covalent bonding from OTC and ZIF-8, as shown on mass spectrometry analysis.

**
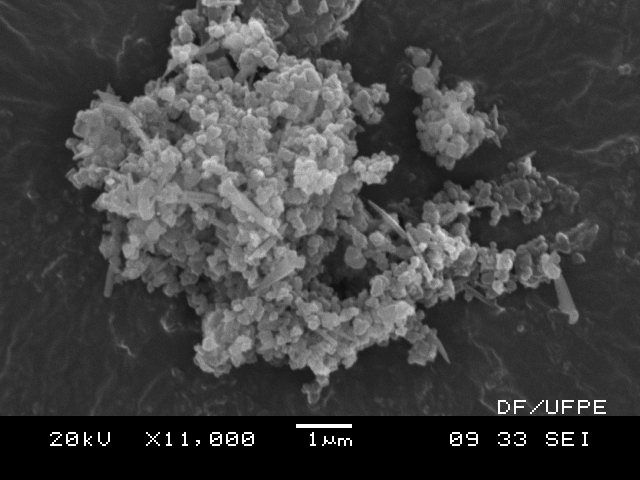
**

***Figure C.*** *SEM micrograph of OTC 40 mg L^-1^ adsorbed on ZIF-8.*

**Thermogravimetric analysis (TGA)**

**
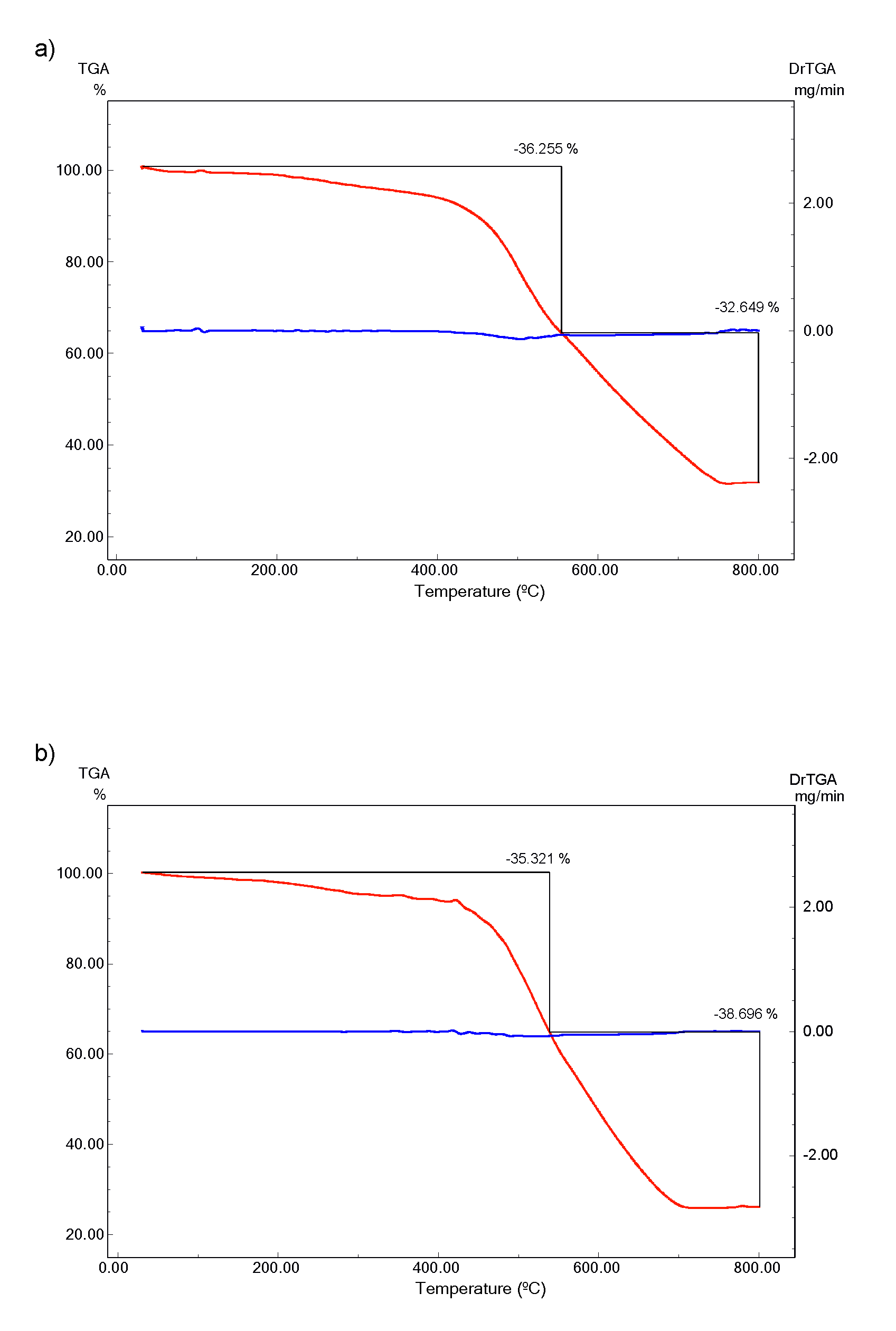
**

***Figure D.****TGA of (a) adsorption product after contact with: 10 mg L^-1^ OTC and (b) 40 mg L^-1^ OTC.*

**Supporting Information Legends**

**Figure A. SEM micrograph of commercial ZIF-8.**

**Figure B. SEM micrograph of OTC.**

**Figure C. SEM micrograph of OTC 40 mg L^-1^ adsorbed on ZIF-8.**

**Figure D.TGA of (a) adsorption product after contact with: 10 mg L^-1^ OTC and (b) 40 mg L^-1^ OTC.**
